# Exploring the Link between Lifestyle, Inflammation, and Insulin Resistance through an Improved Healthy Living Index

**DOI:** 10.3390/nu16030388

**Published:** 2024-01-29

**Authors:** Franz Bruckner, Judith R. Gruber, Alea Ruf, Sharmili Edwin Thanarajah, Andreas Reif, Silke Matura

**Affiliations:** 1 Department of Psychiatry, Psychosomatic Medicine and Psychotherapy, University Hospital, Goethe University Frankfurt, Heinrich-Hoffmann-Str. 10, 60528 Frankfurt am Main, Germany; s2720195@stud.uni-frankfurt.de (F.B.); j.gruber@med.uni-frankfurt.de (J.R.G.); alea.ruf@univie.ac.at (A.R.); edwinthanarajah@med.uni-frankfurt.de (S.E.T.); reif@med.uni-frankfurt.de (A.R.); 2 Fraunhofer Institute for Translational Medicine and Pharmacology ITMP, Theodor-Stern-Kai 7, 60596 Frankfurt am Main, Germany

**Keywords:** Healthy Living Index (HLI), lifestyle, insulin resistance, inflammation, Ecological Momentary Assessment (EMA)

## Abstract

Lifestyle factors—such as diet, physical activity (PA), smoking, and alcohol consumption—have a significant impact on mortality as well as healthcare costs. Moreover, they play a crucial role in the development of type 2 diabetes mellitus (DM2). There also seems to be a link between lifestyle behaviours and insulin resistance, which is often a precursor of DM2. This study uses an enhanced Healthy Living Index (HLI) integrating accelerometric data and an Ecological Momentary Assessment (EMA) to explore differences in lifestyle between insulin-sensitive (IS) and insulin-resistant (IR) individuals. Moreover, it explores the association between lifestyle behaviours and inflammation. Analysing data from 99 participants of the *m*PRIME study (57 women and 42 men; mean age 49.8 years), we calculated HLI scores—ranging from 0 to 4— based on adherence to specific low-risk lifestyle behaviours, including non-smoking, adhering to a healthy diet, maximally moderate alcohol consumption, and meeting World Health Organization (WHO) PA guidelines. Insulin sensitivity was assessed using a Homeostatic Model Assessment of Insulin Resistance (HOMA-IR) and C-reactive protein (CRP) levels were used as a proxy for inflammation. Lifestyle behaviours, represented by HLI scores, were significantly different between IS and IR individuals (U = 1529.0; *p* = 0.023). The difference in the HLI score between IR and IS individuals was mainly driven by lower adherence to PA recommendations in the IR group. Moreover, reduced PA was linked to increased CRP levels in the IR group (r = −0.368, *p* = 0.014). Our findings suggest that enhancing PA, especially among individuals with impaired insulin resistance, holds significant promise as a preventive strategy.

## 1. Introduction

Lifestyle factors such as diet, physical activity (PA), smoking, and alcohol consumption are significantly associated with all-cause mortality [1]. Non-compliance with lifestyle recommendations not only results in individuals suffering from diseases but also poses significant challenges for the healthcare system [2]. Consequently, the examination of modifiable lifestyle factors and their effects is of considerable societal importance, particularly regarding their preventive potential. A possible instrument for measuring lifestyle is the Healthy Living Index (HLI) by Ford et al. [1], which allows the analysis of health behaviours. This HLI encompasses diet, physical activity, smoking, and alcohol consumption [1]. The goal is to capture lifestyle as complexly as necessary and as simply as possible. The multidimensional structure of the index, unlike the isolated examination of individual factors, gives a comprehensive picture of lifestyle behaviours.

The description of lifestyle behaviours using multidimensional indices has become established in scientific practice. Associations between lifestyle indices and cardiovascular diseases [3], dementia [4], and all-cause mortality have been demonstrated [1].

In addition to lifestyle indices that capture factors of individual behaviour, there are also indices that take environmental factors into account. Hobbs et al. [5] identified five positive and four negative environmental influences, which were summarized into a HLI with nine categories. The results indicate that individuals living in a health-promoting environment exhibit better mental health [5]. Besides the effects on mental health, it has been observed, using the example of obesity, that environmental conditions also have an impact on somatic health [6]. However, a universally accepted index that standardizes the components, their measurement methods, and their cut-off values does not exist. Therefore, in this study, we aimed to compose an enhanced HLI based on the index by Ford et al. [1], which integrates nutrition, PA, smoking, and alcohol consumption. Importantly, and unlike all other indices, our HLI incorporates objective data on PA, derived from accelerometry and detailed dietary information obtained through an Ecological Momentary Assessment (EMA). This index could be used as a valuable tool to detect differences in lifestyle between individuals affected by “civilization diseases” such as type 2 diabetes mellitus (DM2) or even before its onset.

DM2 contributes to somatic comorbidities and detrimentally affects individuals’ quality of life and mental health [7]. The standard treatment of DM2 comprises lifestyle modifications [8], including a healthy diet, regular PA, and smoking cessation. Also, moderate alcohol consumption can have a protective effect on the development of DM2 [9]. Previous studies have shown that differences in lifestyle behaviours influence the risk of developing diabetes [10,11,12,13]. In a systematic review, Khan et al. (2023) demonstrated that adherence to multiple low-risk lifestyle behaviours reduces the risk of DM2 by 85% [13]. A study by Schlesinger et al. (2020) revealed a dose-dependent relationship: for each additional lifestyle factor adhered to there is a 32% reduction in diabetes risk [10]. Long et al. (2015) showed that compliance with the four lifestyle recommendations comprising the HLI could prevent 34% of all diabetes cases [12]. Given the rapidly increasing prevalence of DM2 [14], a comprehensive understanding of the mechanisms that lead to the development of DM2 is crucial. This study employs the HLI as a tool to investigate potential disparities in lifestyle between insulin-sensitive (IS) and insulin-resistant (IR) individuals. Insulin resistance, i.e., impaired sensitivity towards insulin, diminishes insulin effectiveness, prompting elevated insulin levels. When the escalating demand for insulin cannot be met, blood sugar levels rise constantly which indicates the progression towards prediabetes and DM2. Therefore, insulin resistance is regarded as a precursor to DM2 [15]. This study marks the first comprehensive examination of lifestyle factors in the at-risk group of IR individuals.

Lifestyle factors, including smoking [16], alcohol consumption [17], poor diet [16,18,19], and low levels of PA [16,20], not only pose a risk for the development of DM2 but can also contribute to the development of low-grade inflammation. Low-grade inflammation can be defined as an increase in systemic plasma concentrations of cytokines like C-reactive protein (CRP) [21]. CRP is widely recognized as the most extensively studied biomarker of chronic inflammation making it highly suitable for measuring inflammation [18]. Chronic inflammatory diseases together with diabetes and cardiovascular diseases represent the foremost cause of global mortality [16]. Apart from the somatic consequences of low-grade inflammation, it also plays a crucial role in psychiatric disorders [22]. So far, only one study has investigated the association between a combination of different lifestyle factors and inflammation [23]. In addition to the factors explored in the current study, M. Sotos-Prieto et al. incorporated social support into the index. The study found a correlation between the HLI of 842 Puerto Ricans in Boston, MA and the levels of Interleukin-6 (IL-6) and Tumour Necrosis Factor-Alpha (TNF-α), but no association was observed between HLI and CRP [23]. Addressing this research gap, we explored the influence of lifestyle on inflammation in IR and IS individuals.

The aim of this study was to investigate whether IR individuals differ from IS individuals in terms of lifestyle. These differences may have contributed to the insulin resistance and in the long-term may lead to DM2. Moreover, we address the question of whether lifestyle, represented by the HLI, is associated with inflammation and study the role of insulin resistance in this association.

## 2. Materials and Methods

### 2.1. Study Design and Participants

The present research is part of the *m*PRIME study, which is a prospective, observational, longitudinal study, based in Frankfurt am Main, Germany. Participants were recruited between March 2021 and March 2023. An initial telephone interview for eligibility screening was conducted. The inclusion criteria of the *m*PRIME study were as follows: all participants were required to be 18 years of age or older and provide written consent for participation. Stringent exclusion criteria were applied in this study, including the use of antidiabetic medications or insulin; a diagnosis of type 1 diabetes mellitus and gestational diabetes; corticosteroid use; various psychiatric diagnoses including bipolar I disorder, schizophrenia, organically caused mental disorders, and substance dependence (other than nicotine and cannabis dependence); severe neurological disorders; pregnancy and breastfeeding; non-correctable visual impairments; participation in medication-related studies within the last 6 months; weight-reducing medications or a diet within the last 3 months; and language barriers. Groups were categorized using the Homeostatic Model Assessment of Insulin Resistance (HOMA-Index) [24], calculated based on fasting glucose and insulin levels [24]. Participants who had a HOMA-Index of ≥2 were assigned to the IR group, while individuals with a HOMA-Index < 2 were assigned to the IS group. To ensure the comparability of these groups, the recruitment strategy specifically focused on individuals with a body mass index (BMI) exceeding 25. The study was approved by the Ethical Committee of Goethe University Hospital Frankfurt. Written informed consent was procured from all study participants.

In the context of the *m*PRIME study, a total of four test appointments were conducted within a year. For the present study, data from the first two appointments were used. The initial on-site test (T1) followed the screening process and involved a comprehensive set of assessments. For this research, the relevant assessments conducted during T1 encompassed the collection of blood samples, the documentation of demographic information and anthropometric measurements, and the administration of neuropsychological tests. During the subsequent week, data on dietary intake and PA were collected through EMA. The second on-site session, T2, took place the following week and involved a debriefing session. Two follow-up sessions were scheduled, one at 3 months post-T1 and another at 12 months post-T1. Data of these follow-up sessions are not subject to this study. At the time of data analysis, all testing sessions were completed, except for the second follow-up.

### 2.2. Data Collection

All assessments were administered by trained staff and participants were provided with a comprehensive introduction to equipment usage for the ambulatory assessment. Gender, age, education level, nationality, marital status, and income were collected by a demographic questionnaire. Height and weight were measured on-site to calculate BMI, which can be determined by dividing body weight (in kg) by the square of body height (in m^2^).

Diet: This study utilized EMA to capture dietary habits through myfood24-Germany (Measure Your Food on One Day) [25]. Using this tool, participants completed a web-based, 24 h dietary recall at T1 and in the subsequent week; three food records were filled out using the mobile version of the tool, ensuring the inclusion of at least one weekend day. We monitored daily calorie intake and conducted a comprehensive analysis of more than 100 different micro- and macronutrients for each day of data collection. Staff reviewed all the recorded protocols and addressed any uncertainties during the second testing session (T2). The dietary data from EMA (myfood24) were used to evaluate participants’ diet quality based on the Healthy Eating Index-NVS (HEI-NVS) [26], which is based on the HEI-1995 [27] and the HEI-EPIC [28]. The HEI-NVS is adapted to German eating habits and assesses nutritional quality using the dietary guidelines of the German Nutrition Society (DGE) [29]. The index consists of 10 components (fruit, vegetables, grains, milk, fish, meat, eggs, spreadable fats, beverages, and alcohol), with a maximum score of 15 for fruits and vegetables and 10 for the remaining categories. In total, a maximum score of 110 points can be achieved in the HEI-NVS. The detailed scoring protocol can be found in the supplementary material (see Supplementary Table S1). During data pre-processing, we included only individuals with a minimum of two dietary protocols. We conducted a stringent assessment of daily calorie intake, resulting in the exclusion of individuals with unrealistic daily energy intake (<600 kcal/day or >6000 kcal/day) [30]. Consequently, seven individuals were excluded from the analysis. For the calculation of the HLI, we excluded the alcohol category since it is incorporated as a separate entity in the HLI, resulting in a maximum score of 100 points for the diet quality. We categorized the dietary data received from myfood24 into the 10 components and computed HEI-NVS scores depending on the consumed quantity. A higher score signifies a closer adherence to the recommendations established by the German Nutrition Society (DGE). According to the index by Ford et al. [1], individuals in the lower 60% of the HEI were assigned a score of zero, while those in the upper 40% received a score of one.

Alcohol: To assess this category, the average daily alcohol consumption was calculated based on the data collected by the food records and dietary recall (see section Diet). Men achieved one point in the HLI if their mean daily alcohol intake remained below 20 g (equivalent to two standard drinks [31]), whereas women received one point if their mean daily alcohol consumption did not exceed 10 g (equivalent to one standard drink [31]). These threshold values align with the guidelines of the German Nutrition Society (DGE) [32] and are consistent with the findings presented in the most recent Federal Health Survey on alcohol published by the Robert Koch Institute (RKI) [33].

Physical Activity: Using the Move3 and Move4 activity sensor (Move 3, Art. No. 10112, from Movisens), we assessed PA, categorizing it based on World Health Organization (WHO) recommendations. Metabolic Equivalents (MET) were calculated using the data analysis software provided by Movisens (DataAnalyzer, version 1.13.7). To calculate the MET, the DataAnalyzer provided by Movisens uses movement acceleration; the altitude change extracted from barometric data; and the personal parameters of age, gender, weight, and height. Participants were instructed to wear the sensor on their non-dominant wrist over 7 days, including during sleep. To categorize PA, we adhered to WHO guidelines, resulting in a dichotomous classification [34]. These recommendations encompass either 150–300 min of moderate or 75–100 min of vigorous activity per week or a combination of both, as defined by an accumulation of >600 MET/week [35]. Activities with a MET value of ≥3 and <6 were categorized as moderate, while those with a MET value of ≥6 were classified as vigorous. The analysis included only individuals who wore the sensor for a minimum of 10 h per day on at least 5 days; consequently, 11 individuals were excluded. Technical difficulties with the sensor resulted in the absence of data for six individuals.

Smoking: Individuals who self-identified as smokers or occasional smokers were assigned a score of zero for the HLI. In contrast, individuals who reported no tobacco use were awarded one point, as aligned with health recommendations.

In summary, the HLI consists of the following variables: diet, PA, smoking, and alcohol consumption. Participants receive either no or one point for each category. The total HLI score for each participant was calculated by aggregating the four category scores, resulting in a final HLI score ranging from zero to four.

Insulin resistance: A venous blood sample was collected in the morning at T1, following a minimum 8 h fasting period for serum analyses conducted on the same day. Serum insulin concentrations were determined using an electrochemiluminescence immunoassay (Roche Diagnostics, Indianapolis, IN, USA, Cobas 8000-e801), while glucose concentrations were measured using the hexokinase method (Roche Diagnostics, Cobas 8000-c701). To assess endogenous insulin sensitivity, the Homeostasis Model Assessment (HOMA) index was calculated as the product of fasting glucose [mg/dL] and fasting insulin [mU/L], divided by 405 [24]. Participants with a HOMA index < 2 were considered IS, while those with a value of 2 or higher were classified as IR.

Inflammation: CRP levels were measured to evaluate inflammation, utilizing a latex-enhanced immunoturbidimetric method (Roche Diagnostics, Cobas 8000-c701), also derived from the serum blood sample.

### 2.3. Statistical Analysis

The statistical analyses were conducted using SPSS Version 29 (SPSS/IBM, Chicago, IL, USA). A significance level of *p* < 0.05 was used for all statistical tests. The data underwent a normality assessment using the Kolmogorov–Smirnov test. Non-parametric tests were used if data was not normally distributed. Categorical variables are represented using the number of individuals (*n*), while for quantitative variables the mean and standard deviation (SD) are provided. Differences between IR and IS individuals were assessed using the Wilcoxon–Mann–Whitney Test, the Kruskal–Wallis Test, the Pearson’s chi-square test (χ^2^), or the independent samples *t*-test. To investigate the association between HLI and CRP, we employed a multiple linear regression to assess whether HLI significantly predicts CRP when controlling for the confounding variables: gender, age, and education. CRP values were log-transformed for all analyses. For HLI categories where IR differed from IS individuals, we examined the association of each category with CRP using partial correlation (controlling for gender, age, and education). This analysis was conducted for the entire cohort as well as for the IR and IS groups separately.

## 3. Results

### 3.1. General Characteristics of Participants

Our analysis includes 99 participants (57 females and 42 males) aged between 18 and 78 years. The initial data set comprised 124 individuals. Data from 25 individuals was either incomplete or of insufficient quality and, therefore, could not be used. Table 1 summarizes the characteristics of all participants and the groups of IR and IS individuals. On average, IR participants were older, had a higher BMI and HbA1c, and attained a lower level of education.

### 3.2. HLI Score and Separate Scores for Smoking, Diet, Alcohol, and PA

The mean values for the HLI and its subcategories, encompassing diet quality, PA, smoking, and alcohol consumption, are presented in Table 2. IS individuals demonstrated higher adherence to WHO recommendations for PA compared to IR individuals (χ^2^ = 15.14, *p* < 0.001), particularly in spending more time engaged in moderate activity (t = −5.945, *p* < 0.001). There was no significant variation between the groups in terms of adherence to the recommendations regarding alcohol consumption (χ^2^ = 0.489, *p* = 0.484) and smoking (χ^2^ = 0.010, *p* = 0.918). There was no significant difference between IS and IR individuals regarding diet quality (t = −1.373, *p* = 0.173).

**Figure 1:**
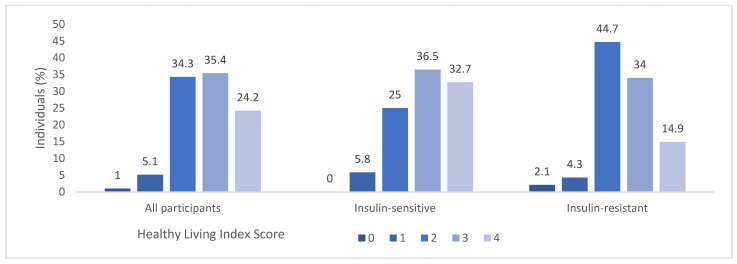
Distribution of Healthy Living Index (HLI) in all participants and in the IS and IR groups. A HLI score of zero signifies lowest adherence to health-promoting lifestyle behaviours whereas a score of four signifies highest adherence to health-promoting lifestyle behaviours.

The examination of HLI factors within the entire cohort as well as broken down for the IR and IS groups are illustrated in Figure 1; significant differences in HLI were observed between IR and IS groups (U = 1529.0; *p* = 0.023) suggesting that higher risk behaviours are found in the IR group. A gender-based analysis using Pearson’s chi-square test found no statistically significant disparities in HLI between men and women (χ2 = 5.776; *p* = 0.217). Additionally, no significant correlation between age and HLI was identified, as indicated by Spearman’s rank correlation coefficient (r_s_ = −0.180; *p* = 0.074).

### 3.3. The Healthy Living Index: Lifestyle and Its Impact on Inflammation

A multiple linear regression model regression revealed that neither gender (β = −0.130, *p* = 0.192), age (β = 0.089, *p* = 0.369), nor education, (β = 0.115, *p* = 0.258) were significant predictors of CRP. However, the HLI exhibited a significant influence on CRP (β = −0.261, *p* = 0.010). This underscores the significant role of HLI in predicting CRP and demonstrates an association between a healthy lifestyle and lower CRP levels (Table 3).

**Figure 2:**
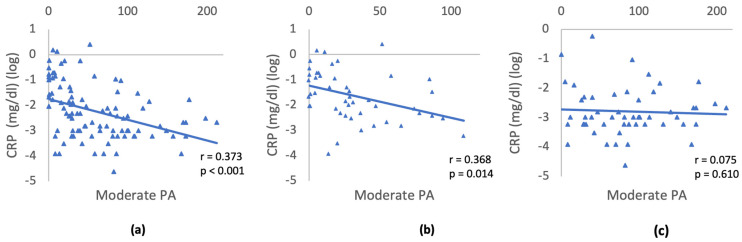
Relationship between CRP and moderate activity controlling for gender, age, and education: (**a**) All participants; (**b**) IR; (**c**) IS individuals. Triangles depict the log transformed CRP values and minutes/day of moderate physical activity for each participant. The slope of the blue line represents the strength and direction of the relationship between the variables CRP and moderate physical activity.

The association between CRP and moderate PA was further explored through partial correlation analysis, adjusting for gender, age, and education (Figure 2). Given the absence of differences between IR and IS individuals in the categories of diet, smoking, and alcohol consumption, no correlation with CRP was conducted. The analysis was conducted separately for the three groups: (1) All participants; (2) Individuals with IR; (3) IS individuals. For all participants, a moderately negative partial correlation was observed between CRP and PA, when controlling for gender, age, and education (r = −0.373, *p* < 0.001). Similarly, within the IR group, a moderately negative partial correlation was found between CRP and PA, controlling for gender, age, and education (r = −0.368, *p* = 0.014). In the IS group no correlation was detected between CRP and PA (r = −0.075, *p* = 0.610). This suggests that reduced PA is significantly linked to higher CRP levels IR individuals, while no significant correlation was evident in the insulin-sensitive group.

## 4. Discussion

This study used a refined HLI to investigate the association between lifestyle behaviours and insulin resistance. Furthermore, the connection between distinct lifestyle factors, including diet, PA, smoking, and alcohol consumption, and inflammation was investigated. Significantly, lower HLI scores and higher CRP values were observed in IR individuals. The differences in HLI were primarily explained by the amount of moderate PA, with IS individuals adhering more closely to the WHO recommendations for PA. In the insulin resistant group, lower levels of moderate physical activity were associated with higher CRP.

Several studies have shown that adherence to a healthy lifestyle significantly reduces the risk of developing DM2 [10,11,12,13]. To date, no study had investigated combined lifestyle behaviours in IR individuals compared to IS individuals. Despite employing a lower threshold for classifying insulin resistance (HOMA ≥ 2) than other studies [36], this study was able to identify differences in lifestyle between IR and IS individuals. These findings suggest that differences in lifestyle might contribute to the development of insulin resistance.

It is important to highlight that the categories in our HLI deviate from those in most reference studies, which frequently incorporate BMI or body weight [10,11,12,13]. We deliberately excluded BMI as there is a strong correlation between increased body weight, reduced PA, and poorer nutrition [37,38]. Thus, we do not consider body weight as an independent lifestyle factor but rather as an outcome of other lifestyle factors. Moreover, there is well-established evidence of the correlation between overweight and insulin resistance [39]. This correlation would introduce bias into our examination of lifestyle factors relevant to insulin resistance. It is also worthwhile to consider the potential advantages of incorporating additional factors (e.g., sleep patterns and social networks), as reported in two meta-analyses, to enhance the precision of evaluating lifestyle elements relevant to insulin resistance [10,13].

Substantial variations in adherence to WHO recommendations for PA between IR and IS individuals were observed, primarily explaining the differences in HLI between the groups. Differences in PA were mainly associated with differences in the duration of time spent engaging in moderate activity. Consistent findings were observed in previous studies, such as the study by Adriouch et al. (2017) that was based on questionnaire data [40]. A study conducted by Ekelund et al. (2009), utilizing accelerometer data, showed an inverse correlation between the duration of time spent performing moderate PA and the HOMA-Index within a sample of 192 individuals [41]. Moreover, the literature provides additional evidence suggesting a positive impact of PA on glucose control in DM2 [42,43,44]. In contrast, divergent findings were observed in a study by Yoshimura et al. which incorporated accelerometer data from a cohort of 12 individuals with DM2 and 10 control participants [45]. This study did not reveal significant differences in moderate activity [45]. Moreover, sedentary behaviour seems to play a crucial role in insulin resistance [46,47]. Decreased PA, as found in our study, is likely to contribute to insulin resistance and eventually contribute to the development of DM2.

In our study, no significant difference in dietary quality between IR and IS individuals was observed. Existing research provides mixed results in this regard. A study with findings consistent with ours employed extensive web-based dietary records and specific dietary quality assessments but observed no differences in the overall dietary index between individuals with and without diabetes. However, when distinct dietary categories were analysed, Adriouch et al. (2017) found that individuals with DM2 consume more processed and red meat, more poultry and vegetables, and fewer fruits and sweet products [40]. Since we did not differentiate between these dietary categories and we did not find differences in diet quality between IR and IS individuals. Future research could investigate variations within specific food categories to gain a more comprehensive understanding of dietary differences between IR and IS individuals.

A study by Castetbon et al. (2006) showed improved dietary habits in individuals with DM2 [48]. One possible explanation is that participants adjusted their previously less healthy dietary habits to dietary recommendations following their diabetes diagnosis. Therefore, individuals with diabetes may initially have poorer dietary habits, which is supported by a subsequent study demonstrating a reverse correlation between insulin resistance and dietary quality [49]. This study found that an increase in the HEI was associated with a 0.5-unit reduction in the HOMA-Index. If we extrapolate the results of these two studies to our cohort, we would have expected to find poorer diet quality in the IR group, as most individuals in our sample were unaware of their insulin resistance. One potential explanation for the absence of these differences could be selection bias in that IR individuals who participated in our study may have exhibited a heightened level of health interest. Furthermore, the awareness of participating in a study may have led to dietary adjustments during the monitoring period.

No differences in smoking habits were observed between IR individuals and IS individuals. Previous studies have found differences in smoking habits between individuals with diabetes and those without diabetes, with smokers having a 45% higher risk of developing diabetes compared to non-smokers [50,51]. Discrepancies between these studies and our results could be attributed to the absence of information on the smoking history of the participants. Former smokers still have an increased risk of insulin resistance [52,53] but were categorized as non-smokers in our data collection, leading to misclassification. Furthermore, it could be beneficial to record the quantity of cigarettes smoked to account for the dose-dependent relationship between the number of cigarettes smoked and the development of insulin resistance [50]. Since we did not assess the smoking history and the quantity of cigarettes smoked, we might have missed potential differences between the groups in smoking habits.

While certain studies have also presented no differences between IR and IS individuals regarding alcohol consumption, other investigations, including a meta-analysis, provide substantial evidence suggesting that moderate alcohol consumption can enhance insulin sensitivity and, consequently, reduce the risk of diabetes [9,54,55,56]. Nevertheless, the incorporation of moderate alcohol consumption as a recommended lifestyle factor in the HLI remains a topic of controversy and some studies raise doubts about the positive effects of alcohol consumption on the cardiovascular system [57]. The WHO also highlights the absence of safe alcohol consumption [58,59]. One plausible explanation for the absence of differences in our study could be the limited assessment of excessive alcohol consumption, a factor significantly associated with an elevated risk of insulin resistance [9,56].

Significant disparities in CRP levels were found between IR and IS individuals. The higher CRP levels in IR are likely to be associated with being overweight. This assumption is supported by existing studies emphasizing the contributory role of increased adipose tissue to elevated CRP levels [60]. In the existing literature, only one study has been identified that investigated the association between lifestyle and inflammatory markers. This study by Sotos-Prieto et al. demonstrated significant correlations of the HLI with TNF-alpha and IL-6; however, no correlation with CRP was observed [23]. Therefore, our investigation aligns with the existing literature and extends it by establishing the association of HLI with CRP. However, previous studies have identified correlations between CRP and individual HLI components. For instance, two studies revealed inverse correlations between CRP and a healthy diet [18,19]. Another study identified positive correlations between CRP, smoking, and alcohol consumption, along with a negative correlation with increased PA [17]. Additionally, one study reported elevated CRP levels associated with low PA [20]. Moreover, a study by Sotos-Prieto et al. (2016) showed an association between PA and sedentary behaviour with IL-6. However, this association disappeared after adjusting for other lifestyle variables [23]. In our study, the variation in the HLI was primarily attributed to differences in PA. Consequently, we specifically employed this category to investigate the correlation between lifestyle and inflammation in both groups separately through partial regression. It was observed that more PA was only linked to reduced CRP levels in the IR group, whereas no correlation between PA and CRP could be identified in the IS group. As visceral fat is significantly associated with heightened inflammatory activity [60], one possible explanation could be that the reduction in adipose tissue, linked to lower inflammation, can be achieved through increased PA. In the study by Villegas et al. (2012), the link between CRP and PA was no longer significant after adjusting for BMI [17]. Geffken et al. suggested that BMI serves as a mediator, providing an explanation for both decreased PA and heightened levels of CRP [61]. The absence of the effect in IS individuals could be attributed to differing biophysiological mechanisms in IR individuals, influencing inflammatory processes through PA. Additionally, the lower values and variances in CRP in IS individuals could contribute to the missing correlation between CRP and PA. The limited sample size might also explain the absence of correlation, as smaller differences are often more apparent in larger cohorts with increased statistical power. To further investigate this relationship, high-resolution (hs) CRP assays or the inclusion of other inflammatory markers could be meaningful. The identification of an inverse correlation between PA and CRP in the IR group underscores the relevance of increased PA, especially for IR individuals, to improve insulin sensitivity.

In comparison to the overall German population, the cohort of the present study has more favourable values in the categories of PA and smoking but less favourable values concerning alcohol consumption and nutrition [62,63,64,65]. Comparing the HLI values in our cohort with values from other studies, including those conducted in countries other than Germany, reveals that a higher number of participants in our cohort achieve high index scores [4,66,67,68]. This may suggest that the relatively high effort associated with study participation has attracted individuals with a greater health-consciousness and relatively higher adherence to lifestyle recommendations.

In addition to the aforementioned limitations, such as the sample size of the study, the absence of identifying binge drinkers, former smokers, and sedentary behaviours, it is essential to consider some additional constraints in our study. Firstly, it is crucial to acknowledge methodological limitations in data collection, including the limited applicability of activity sensors in certain situations, such as swimming pools or volleyball, where relevant movements occur but cannot be accurately measured. Potential computer-related issues in using myfood24 could have biased data collection. The impact on the results would be negligible if these methodological challenges occurred uniformly for all participants. Secondly, the determination of thresholds for smoking, alcohol consumption, and PA was based on absolute values, while the nutritional threshold within the cohort was created relative to the cohort itself. Given the nutritional bias in the present cohort, this may lead to individuals with unhealthy dietary habits being erroneously classified as “healthy”, complicating the interpretation of the results. Third, the presence of potential unmeasured confounding factors, including genetic factors, medications, or other pre-existing conditions that explain inflammation or insulin resistance, is worth noting. Fourth, the HLI relies on the dichotomization of variables, leading to a loss of information.

This study has also several notable strengths, including the integration of accelerometer data for the objective assessment of PA and the use of EMA for more accurate recording of dietary habits. These factors contribute to the HLI proving suitable for capturing nuanced differences in lifestyle, despite the dichotomization of variables. So far, most studies have investigated lifestyle factors in individuals with and without DM2. Our study is among the very few studies that have focused specifically on the vulnerable group of IR individuals, thereby adding to our understanding of the mechanisms that might contribute to the development of DM2.

## 5. Conclusions

We showed that insulin resistance is linked to a lower adherence to health promoting lifestyle behaviours. Insulin-sensitive individuals showed significantly higher HLI scores than insulin-resistant individuals. The difference in HLI scores is primarily due to reduced levels of physical activity in insulin-resistant individuals.

Additionally, our study showed a negative correlation between adherence to lifestyle recommendations and inflammation. More specifically, inflammation was linked to reduced levels of physical activity in insulin-resistant individuals. Given the connection between physical activity and inflammation in the insulin-resistant group, incorporating more physical activity into the daily lives of individuals at risk of developing type 2 diabetes (DM2) holds promise as a preventive measure.

## Figures and Tables

**Table 1:** General characteristics of the final sample of the present study.

	All	IR	IS	*p*-Value
	*n* = 99	*n* = 47	*n* = 52	
Gender *n*, (%)				0.430 ^a^
Women	57 (57.6)	29 (61.7)	28 (53.8)	
Men	42 (42.4)	18 (38.3)	24 (46.2)	
Age (years, mean ± SD)	49.8 (14.1)	53.2 (11.6)	46.8 (15.5)	0.022 * ^b^
Highest education attainment *n*, (%)				0.047 * ^b^
Secondary school	1 (1)	1 (2.1)	0	
Certificate of Secondary Education ^c^	8 (8,1)	7 (14.9)	1 (1.9)	
High School Diploma ^d^	11 (11,1)	4 (8.5)	7 (13.5)	
Completed vocational training	20 (20.2)	12 (25.5)	8 (15.4)	
Degree from University of Applied Sciences	7 (7.1)	3 (6.4)	4 (7.7)	
Bachelor’s degree	7 (7.1)	2 (4.3)	5 (9.6)	
Master’s degree	39 (39.4)	16 (34)	23 (44.2)	
Ph.D.	6 (6.1)	2 (4.3)	4 (7.7)	
HbA1c (%; mean ± SD)	5.5 (0.4)	5.6 (0.4)	5.3 (0.3)	<0.001 ** ^b^
BMI (kg/m^2^; mean ± SD)	28.2 (6.2)	32 (6.3)	24.4 (2.7)	<0.001 ** ^b^

^a^ Pearson’s chi-square test (χ^2^), ^b^ Wilcoxon–Mann–Whitney Test; SD: standard deviation; IR: insulin-resistant; IS: insulin-sensitive; ^c^ Certificate of Secondary Education—the American equivalent of mittlere Reife in Germany; ^d^ the American equivalent of Abitur in Germany; BMI: body mass index; HbA1c: a measure of the average blood sugar level; * *p* < 0.05; ** *p* < 0.01.

**Table 2:** Differences in HLI and the single categories between the groups of IS and IR individuals.

	All	IR	IS	*p*-Value
	*n* = 99	*n* = 47	*n* = 52	
HLI Score (mean ± SD)	2.7 (0.9)	2.5 (0.9)	2.9 (0.9)	0.023 ^b^ *
HEI Score (mean ± SD)	60.2 (9.3)	58.8 (10.2)	61.4 (8.3)	0.173 ^c^
Moderate PA (min/day) (mean ± SD)	59.1 (51.8)	31.2 (29.3)	84.5 (54.8)	<0.001 ^c^
Meeting WHO-rec. for PA *n*, (%)	71 (71.7)	25 (53.2)	46 (88.5)	<0.001 ** ^a^
Meeting alcohol rec. *n*, (%)	77 (77.8)	38 (80.9)	39 (75)	0.484 ^a^
Meeting smoking rec. *n*, (%)	86 (86.9)	41 (87.2)	45 (86.5)	0.918 ^a^
CRP (mg/dL), median (IQR)	0.09 (0.17)	0.19 (0.33))	0.05 (0.06)	<0.001 ** ^b^

IR: insulin-resistant individuals; IS: insulin-sensitive individuals; *n*: number of participants; SD: standard deviation; HLI: Healthy Living Index; HEI: Healthy Eating Index; Moderate; PA: mean time in minutes per day of moderate physical activity, measured as Metabolic Equivalent of Task (MET) values of ≥3 and <6; WHO: World Health Organization; rec: recommendations; CRP: C-reactive protein; * *p* < 0.05; ** *p* < 0.01. ^a^ Pearson’s chi-squared test (χ^2^). ^b^ Wilcoxon–Mann–Whitney Test. ^c^ Independent samples *t*-test.

**Table 3:** Regression model: predicting CRP using gender, age, education, and HLI as independent variables in all participants.

Effect	β	SE	95% CI		*p*
			LL	UL	
Gender	−0.130	0.093	0.063	−0.307	0.192
Age	0.089	0.003	0.009	−0.004	0.369
Education	−0.115	0.025	0.021	−0.077	0.258
HLI	−0.261	0.051	−0.032	−0.235	0.010 *

SE: standard error of the unstandardized coefficient; β: standardized coefficient; CI = confidence interval; LL = lower limit; UL = upper limit; HLI: Healthy Living Index; * *p* < 0.05.

## Data Availability

The data presented in this study are available on request from the corresponding author. The data are not publicly available due to privacy and ethical concerns.

## Supplementary Materials

The following supporting information can be downloaded at: https://www.mdpi.com/article/10.3390/nu16030388/s1; Table S1: Scoring System of the Healthy Eating Index of the German National Nutrition Survey II (HEI-NVS).
