# Supplementary material for: Exploring the Link between Lifestyle, Inflammation, and Insulin Resistance through an Improved Healthy Living Index
Source: Nutrients. 2024 Jan 29;16(3):388. doi: 10.3390/nu16030388 (PMC10857191; doi:10.3390/nu16030388)
Supplement: Supplementary file 1 [file nutrients-16-00388-s001.zip › nutrients-2810271-supplementary.pdf]

Table S1

Scoring System of the Healthy Eating Index of the German National Nutrition Survey II (HEI-NVS)

Category	Foods	Recommendation	Scoring	
Fruits	Raw and cooked fruit, fruit products (e.g., fruit sauces), nuts, seeds	250 g per day (=2 portions): 200 ml of fruit juice and 25 g of seeds and/or nuts[a] can substitute one portion each, but not more	x * 10 / 250 (max. 15 points)	
Vegetables	Raw and cooked vegetable, lettuce, pulses, vegetable-based dishes	400 g per day (=3 portions): 200 ml of vegetable juice can substitute one portion, but not more	x * 10 / 400 (max. 15 points)	
Grains	Grain products such as bread, baked goods, cereals, pasta, potatoes, grain-based dishes	350 to 560 g per day	*If x = 350-560 g:*	10 points
			If x ≤ 350 g:	x * 10 / 350
			If x > 560 g:	560 * 10 / x
Milk	Milk, milk products such as cheese, yoghurt, curd, milk-based dishes	2 portions per day: 1 portion = 200 to 250 g milk/yoghurt or 50 to 60 g cheese/curd[b]	*If x = 400-500 g*:	10 points
			If x ≤ 400 g:	x * 10 / 400
			If x > 500 g:	500 * 10 / x
Fish	Fish, fish products, fish-based dishes	150 to 220 g per week	*If x = 150-220 g:*	10 points
			If x ≤ 150 g:	x * 10 / 150
			If x > 220 g:	220 * 10 / x
Meat	Meat, meat products, sausages, meat-based dishes	< 300 to 600 g per week	*If x ≤ 300 g:*	10 points
			If x > 600 g:	600 * 10 / x
Egg	Eggs and egg-based dishes	≤ 3 eggs per week (= 180 g)	*If x ≤ 180 g:*	10 points

Category	Foods	Recommendation	Scoring	
			If x > 180 g:	180 * 10 / x
Alcohol	Pure alcohol (i.e., ethanol)	Women: ≤ 10 g per day	*If x ≤ 10/20g:*	10 points
		Men: ≤ 20 g per day	*If x > 10/20g:*	30 * 10 / x
Spreadable fats	Butter, margarine	≤ 15 to 30 g per day	*If x ≤ 15 g:*	10 points
			If x > 30 g:	30 * 10 / x
Drinks	Alcohol-free drinks such as water, coffee, tea, fruit juice and nectar, vegetables juice, lemonade	≥ 1.5 liters per day	*If x ≥ 1.5 liters:*	10 points
			If x < 1.5 liters:	x * 10 / 1.5

Note. x = actual intake.

[a] According to the 10 guidelines of the German Nutrition Society (DGE), 25 g of seeds and/or nuts can substitute one portion of fruits and were therefore included in the fruit category.

[b] To streamline the parallelization of the two subcategories, the amount of the subcategory cheese/curd was transferred to the subcategory milk/yoghurt by multiplying it by 4. As a result, 400 to 500 g could be used as the recommendation of two portions keeping the portion discrepancy between the two subcategories minimal.
